# Assessment of Barriers and Enablers to Implementation of a Pediatric Early Warning System in Resource-Limited Settings

**DOI:** 10.1001/jamanetworkopen.2022.1547

**Published:** 2022-03-09

**Authors:** Asya Agulnik, Gia Ferrara, Maria Puerto-Torres, Srinithya R. Gillipelli, Paul Elish, Hilmarie Muniz-Talavera, Alejandra Gonzalez-Ruiz, Miriam Armenta, Camila Barra, Rosdali Diaz, Cinthia Hernandez, Susana Juárez Tobias, Jose de Jesus Loeza, Alejandra Mendez, Erika Montalvo, Eulalia Penafiel, Estuardo Pineda, Dylan E. Graetz

**Affiliations:** 1 Department of Global Pediatric Medicine and Division of Critical Care, St Jude Children’s Research Hospital, Memphis, Tennessee; 2 Department of Global Pediatric Medicine, St Jude Children’s Research Hospital, Memphis, Tennessee; 3 College of Medicine, Baylor University, Houston, Texas; 4 Rollins School of Public Health, Emory University, Atlanta, Georgia; 5 Department of Pediatric Oncology, Hospital General de Tijuana, Tijuana, México; 6 Department of Pediatric Oncology, Hospital Dr Luis Calvo Mackenna, Santiago, Chile; 7 Instituto Nacional de Enfermedades Neoplásicas, Lima, Perú; 8 Department of Pediatric Oncology, Hospital Infantil Teletón de Oncología, Querétaro, México; 9 Hospital Central Dr Ignacio Morones Prieto, San Luis Potosí, México; 10 Department of Pediatric Oncology, Hospital Centro Estatal de Cancerología, Xalapa, México; 11 Department of Pediatric Critical Care, Unidad Nacional de Oncología Pediátrica, Guatemala City, Guatemala; 12 Department of Pediatric Critical Care, Hospital Oncológico Solca Núcleo de Quito, Quito, Ecuador; 13 Department of Pediatric Oncology, Instituto del Cáncer SOLCA Cuenca, Cuenca, Ecuador; 14 Department of Pediatric Oncology, Hospital Nacional de Niños Benjamín Bloom, San Salvador, El Salvador

## Abstract

**Importance:**

Pediatric early warning systems (PEWS) aid with early identification of clinical deterioration and improve outcomes in children with cancer hospitalized in resource-limited settings; however, there may be barriers to implementation.

**Objective:**

To evaluate stakeholder-reported barriers and enablers to PEWS implementation in resource-limited hospitals.

**Design, Setting, and Participants:**

In this qualitative study, semistructured stakeholder interviews were conducted at 5 resource-limited pediatric oncology centers in 4 countries in Latin America. Hospitals participating in a multicenter collaborative to implement PEWS were purposefully sampled based on time required for implementation (fast vs slow), and stakeholders interviewed included physicians, nurses, and administrators, involved in PEWS implementation. An interview guide was developed using the Consolidated Framework for Implementation Research (CFIR). Interviews were conducted virtually in Spanish, audiorecorded, and professionally transcribed and translated into English. A codebook was developed a priori using the CFIR and supplemented with codes inductively derived from transcript review. Two coders independently analyzed all transcripts, achieving a κ of 0.8 to 0.9. The study was conducted from June 1 to August 31, 2020.

**Main Outcomes and Measures:**

Thematic analysis was conducted based on CFIR domains (inner setting, characteristics of individuals, outer setting, intervention characteristics, and implementation process) to identify barriers and enablers to PEWS implementation.

**Results:**

Seventy-one staff involved in PEWS implementation were interviewed, including 32 physicians (45%), 32 nurses (45%), and 7 administrators (10%). Of these, 50 were women (70%). Components of the 5 CFIR domains were mentioned by participants as barriers and enablers to PEWS implementation at both fast- and slow-implementing centers. Participants emphasized barriers at the level of the clinical staff, hospital, external factors, and PEWS intervention. These barriers included staff resistance to change, inadequate resources, components of health systems, and the perceived origin and complexity of PEWS. At all centers, most barriers were successfully converted to enablers during the implementation process through targeted strategies, such as early stakeholder engagement and adaptation, including adapting PEWS to better fit the local context and changing the hospital setting to support ongoing use of PEWS.

**Conclusions and Relevance:**

To date, this is the first multicenter, multinational study describing barriers and enablers to PEWS implementation in resource-limited settings. Findings suggest that many barriers are not immutable and can be converted to enablers during the implementation process. This work can serve as a guide for clinicians looking to implement evidence-based interventions to reduce global disparities in patient outcomes.

## Key Points

**Question:**

What barriers and enablers to pediatric early warning systems (PEWS) implementation in resource-limited hospitals are reported by health care professionals?

**Findings:**

In this qualitative study including 5 resource-limited pediatric oncology centers in 4 countries in Latin America, many barriers to PEWS implementation were identified, including inadequate resources and staff resistance to change. Most barriers were successfully converted to enablers during implementation through strategies such as early stakeholder engagement, adapting PEWS to the local context, and changing the hospital setting to support use of PEWS.

**Meaning:**

The findings of this study suggest that barriers to implementation of evidence-based interventions in resource-limited settings are not immutable and can be converted to enablers through targeted implementation strategies.

## Teaser

This qualitative study examines issues reported by health care professionals regarding implementation of pediatric early warning systems in resource-limited pediatric oncology facilities in 4 Latin American countries.

## Introduction

Prevention and management of critical illness are integral to improve survival for children globally, particularly for those at high risk for clinical deterioration, such as children with cancer.^1^ However, resources for pediatric critical care vary worldwide, including limitations to funding, equipment, medicine, physical space, and staff needed to provide optimum patient care.^2^ Pediatric early warning systems (PEWS) comprise bedside assessment tools associated with an action algorithm for early identification of deterioration.^3,4,5^ PEWS have been validated to identify critical illness,^6,7^ including in children with cancer.^8,9^ Implementation of PEWS improves patient outcomes, reduces the cost of care, and optimizes interdisciplinary communication in resource-limited hospitals.^10,11,12,13,14,15,16^

Despite evidence of their benefits, PEWS are not widely used in hospitals with resource limitations.^5^ Although reasons for this practice gap are multidimensional, resource-limited hospitals likely experience specific barriers to implementation of evidence-based practices, and challenges implementing PEWS may discourage their use in these settings. A deeper understanding of the barriers and enablers to PEWS implementation may help guide strategies to overcome these challenges. However, to our knowledge, the current literature is limited to single-institution studies conducted in high-income countries.^17,18,19,20,21^ To reduce global disparities in patient outcomes, research is needed to understand factors affecting implementation of interventions such as PEWS in resource-limited settings.

The Consolidated Framework for Implementation Research (CFIR) is used to describe factors associated with successful implementation of evidence-based practices across 5 domains: inner setting, characteristics of individuals, outer setting, intervention characteristics, and implementation process,^22,23^ with evidence supporting its use in resource-limited settings.^24^ This study used the CFIR framework to evaluate barriers and enablers to PEWS implementation in resource-limited hospitals in Latin America and explore strategies that support implementation in these settings.

## Methods

### Escala de Valoración de Alerta Temprana

Escala de Valoración de Alerta Temprana (EVAT) is a Spanish-language PEWS validated in children with cancer.^9^ Proyecto EVAT is a collaborative led by St Jude Children’s Research Hospital (St Jude), which has supported PEWS implementation at more than 40 pediatric oncology hospitals in Latin America.^25,26^ Hospitals are recruited through collaboration with the St Jude Global Alliance^26^ or via learning about the program from colleagues. These centers self-identify as resource-limited owing to challenges including inadequate nursing and physician staff, limited equipment and physical space, and patients with low socioeconomic, educational, and nutritional indicators.^27,28,29,30^ New Proyecto EVAT centers assemble a multidisciplinary implementation leadership team of physicians and nurses who are mentored by St Jude and regional PEWS experts through a standardized implementation process, including planning, training staff, piloting, implementation, and assessment of outcomes. Although all hospitals follow the same process, centers have required variable time (range, 3-12 months) to achieve high-quality PEWS use and complete PEWS implementation.^31^

This study was approved by the institutional review board of St Jude as an exempt, minimal risk study. Additional approvals were obtained by participating centers as needed. As an exempt study, written participant consent was waived; verbal consent was provided at the start of each interview. The Consolidated Criteria for Reporting Qualitative Research (COREQ) reporting guideline was followed to ensure rigor of qualitative reporting.

### Site and Participant Selection

To evaluate barriers and enablers to PEWS implementation, centers were purposefully sampled to include those that implemented PEWS quickly (3-4 months between pilot start and implementation completion) and those that took longer (10-11 months), aiming for regional representation from Mexico, Central America, and South America. All centers completed PEWS implementation before the start of the COVID-19 pandemic (ie, before March 2020). Each center selected a study lead who identified 10 to 15 participants who were (1) implementation leaders (physicians and nurses responsible for local implementation of PEWS), (2) hospital directors (clinicians or administrators with a leadership role in the hospital), or (3) indirectly involved in PEWS implementation. This target sample was chosen as the estimated number needed to reach thematic saturation at each center.^32^

### Interview Methods

The interview guide (eFigure in the Supplement) was developed using the CFIR^22,23^ with adaptations suggested for resource-limited settings.^24^ The preliminary guide was translated to Spanish, iteratively edited by the study team for relevancy and comprehension, piloted with 3 individuals from centers not participating in this study but representative of target participants, and revised based on feedback.

Interviews were conducted in Spanish via a video conferencing platform (WebEx; Cisco) by bilingual members of the study team (S.R.G. and P.E.) from June 1 to August 31, 2020. The interviewers were not previously known to the participants, did not work in their center, and were not involved in PEWS implementation. Interviews were audiorecorded, professionally transcribed and translated into English, and deidentified for analysis.

### Data Analysis

A codebook (eTable 1 in the Supplement) was developed using a priori codes from the CFIR^22^ and novel codes derived inductively by 2 of us (A.A. and G.F.) through review of 9 transcripts. Two of us (A.A. and G.F.) independently coded all transcripts using MAXQDA software (VERBI Software GmbH). These coders ultimately achieved a κ of 0.8 to 0.9 and met regularly to resolve discrepancies through consensus with a third team member (D.E.G.). Thematic content analysis focused on identifying barriers and enablers to PEWS implementation using the CFIR domains (eTable 2 in the Supplement), with constant comparative analysis across transcripts.^33^ Identified themes were compared across slow- and fast-implementing centers.

## Results

Five pediatric oncology centers in 4 countries in Latin America (Ecuador, El Salvador, Mexico, and Peru) were included (eTable 3 in the Supplement). These centers underwent a similar PEWS implementation process but differed in their hospital type, funding structure, pediatric oncology volume, oncology and intensive care unit (ICU) service organization, and time required for implementation. We interviewed 71 participants (Table 1), including physicians (32 [45%]), nurses (32 [45%]), and administrators and data managers (7 [10%]); 21 men (30%) and 50 women (70%) were included.

**Table 1.** Characteristics of 71 Interview Participants

Characteristic	No. (%)
Center	
Lima, Peru	18 (25.4)
San Luis Potosi, Mexico	11 (15.5)
San Salvador, El Salvador	15 (21.1)
Cuenca, Ecuador	15 (21.1)
Xalapa, Mexico	12 (16.9)
Profession	
Floor physician	26 (36.6)
ICU Physician	6 (8.5)
Nurse	32 (45.1)
Other	7 (9.9)
Sex	
Male	21 (29.6)
Female	50 (70.4)
Years working in center	
0-10	27 (38.0)
11-20	25 (35.2)
≥21	19 (26.8)
Role in hospital	
Administrator	8 (11.3)
Clinician	30 (42.3)
Clinician-director	33 (46.5)
Role in PEWS implementation	
Implementation leader	39 (54.9)
Director	21 (29.6)
Other	11 (15.5)

Abbreviations: ICU, intensive care unit; PEWS, Pediatric Early Warning System.

Identified barriers to PEWS implementation were similar across all 5 institutions and involved all CFIR domains; however, most were converted to enablers during the PEWS implementation process. The Figure describes a modified CFIR framework based on identified themes.

**Figure.**
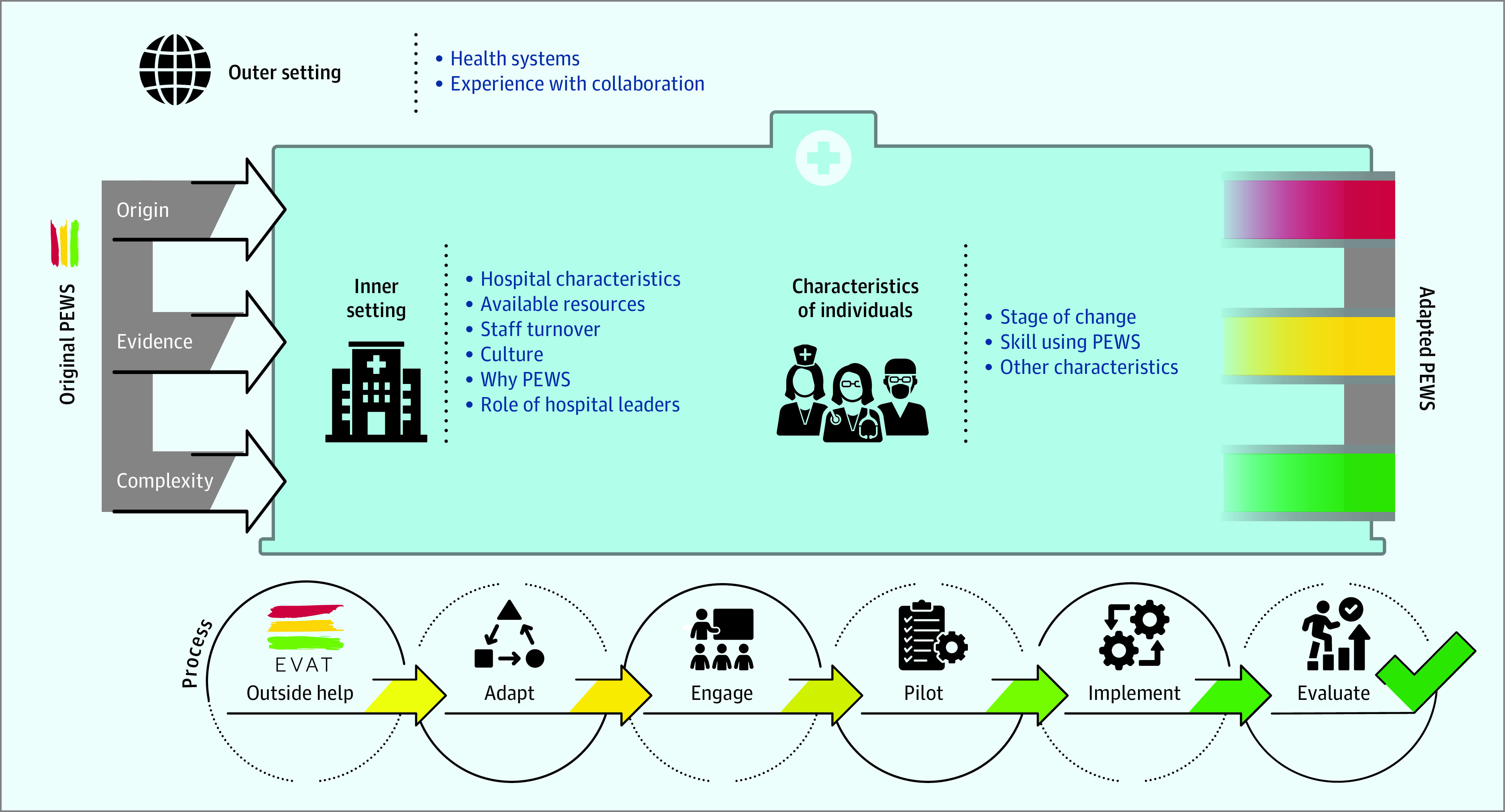
Modified Consolidated Framework for Implementation Research (CFIR) Describing Identified Themes Through the pediatric early warning systems (PEWS) implementation process, centers were able to overcome identified barriers and adapt both the PEWS tool and algorithm as well as their hospital context to support ongoing PEWS use. EVAT indicates Escala de Valoración de Alerta Temprana.

### Inner Setting

Elements of the inner setting contributing to PEWS implementation included hospital characteristics, available resources, staff turnover, the role of hospital leaders, culture, and an understanding of why PEWS was needed. Examples of how these factors manifested as barriers or enablers are provided in Table 2.

**Table 2.** Barriers and Enablers to PEWS Implementation

Domain	Theme	Example	
		Barrier	Enabler
Inner setting	Hospital characteristics	We’re a hospital of specialties, we treat more than 80 specialties and subspecialties; it’s difficult for them to be involved in all areas and in all projects (quality director, San Luis Potosi)	The implementation process of PEWS at [our hospital]...was faster, in a more organized way, we felt the support of everyone, maybe because it was a smaller hospital with fewer staff to organize and commit (implementation leader, Cuenca)
		And also, the extra workload, not because of the project, but we’re a center that unfortunately is having more patients each time, from last year to this year, we experienced 20% to 30% increase in the flow of patients; with the same amount of staff, it was difficult (implementation leader, Xalapa)	Since we are an oncology hospital, we’ve always considered ourselves as a special hospital. In this state, we have many general hospitals and with specialties, but we’re sure we’re different; this is why we try to be updated and have good reception for those programs that strengthen our patient’s safety (nurse director, Xalapa)
	Material resources	The main barriers [were] not having enough supplies to take vital functions properly or monitors, for example (implementation leader, Lima)	Yes, I think enough resources, adapted to our reality; for example, we had snacks during trainings and educator helped with the educational materials (nurse director, Cuenca)
		Because it’s a hospital with multiple specialties, the ICU is not exclusive for oncology, we share it with the other specialties, so sometimes the lack of space in the ICU is still an issue (implementation leader, San Salvador)	It’s never enough when we talk about resources. We’d love to have more, maybe to have monitors, more equipment….But it was enough to start with (implementation leader, Xalapa)
	Human resources	The nurse’s time at the hospital is very limited…the nurse-patient [ratio] is 6:1, and at the medical center is 9-10 patients for every nurse. It was a huge challenge to try to implement a project when the nurse-patient [ratio] is not the right one (implementation leader, San Salvador)	The human resources that we have here, doctors, nurses, [were enough] to facilitate the direct communication and continuous training for the staff (implementation leader, Cuenca)
	Culture	Like we say here in Mexico, the staff has many tricks. They are used to do certain things, even though we know those are not the right ways to do it, and that has generated or may generate in certain moments a barrier for new ideas (implementation leader, San Luis Potosi)	This hospital is in constant [growth]…we are constantly trying, for the sake of the patient, to find the best technologies, the best trainings; there are investigation projects that are always running here (research director, Cuenca)
		What we saw at the beginning was that doctors put a little bit of resistance because very often we have that culture of I'm the doctor, and I'm the one who decides so we don’t allow anyone to tell us what to do (implementation leader, San Salvador)	I think the fact that it was started in pediatrics, that is a compact team, more united. I think that might have helped. But to make it faster…I think it was because of the nurses’ participation and that pediatrics is a united team inside the hospital (implementation leader, Xalapa)
	Role of hospital leaders	The chief of nursing would put barriers and if she was doing that so the rest of the nurses would never feel this was something they should do (physician director, San Luis Potosi)	Once we had the support of the chiefs, it was part of our daily work and that’s how we managed the whole team to participate...finding the support of the chiefs and the institution. I think that pushed the project forward (implementation leader, Lima)
Characteristics of individuals	Stage of change	I think the biggest barrier was the change of thinking. It’s not easy for the Salvadorian who has always been walking in the right side of the road to suddenly change and say to them, now walk on the left side (quality improvement coordinator, San Salvador)	The entire staff knew what was PEWS and what PEWS implied, so it was something very beautiful they already considered PEWS a part of the institution, part of the routine, something we had to do (implementation leader, Lima)
		I think every change generates rejection, to take something out of their comfort zone, something they already have in their nurse routine to add a change, it always generates rejection (implementation leader, San Luis Potosi)	The staff’s acceptance, the willingness to implement the project, the dedication. It was an absolute dedication, in time, in study, in training…it was a time when people got very motivated and I think that was very helpful (implementation leader, Cuenca)
	Skill using PEWS	There are many mistakes and errors we can make as nurses in the evaluation of the patients, or maybe we learned once in the university and we haven’t applied it again (implementation leader, Cuenca)	We see that sometimes we don’t do the evaluations correctly, with practice, we get to see those details and as far as I can see, the staff now applies the evaluation in an objective manner, there are very few mistakes now, almost none (implementation leader, Xalapa)
	Other characteristics	The staff is used to do things their own way since 20 or 30 years and they are not open to new ideas to improve the service (implementation leader, San Luis Potosi)	I think [it] is a hospital characterized by having a staff with high sensitivity and empathy with patients, so, they’re always looking for challenges and improvements, so patients can receive a better attention (research director, Xalapa)
		When we started the implementation of PEWS it was big challenge because I was conscious that some of them never rotated in pediatrics, they were not experts in pediatrics (nurse director, Lima)	I think young people are more open minded to be able to learn and implement new things. I think it facilitates the implementation of this and other programs (physician director, San Luis Potosi)
Outer setting	Health systems	We’re a third-world country, we don’t have enough funds for health and looking for a way to save money for the hospital (implementation leader, San Salvador)	In our country Peru, the morbimortality, especially in pediatric patients, is a national problem, I think that has been the motivation, I think all of this has intervened (physician director, Lima)
	Experience collaboration		One of the assistant doctors that works with us, a rotation she did in Boston, she took this idea and made it happen based on the experiences from other places (physician director, Lima)
PEWS characteristics	Origin	Well, at the beginning they didn’t believe in the program, they thought it was a 1-person program that would benefit 1 person [physician leader] (nurse director, San Luis Potosi)	Being a project endorsed by St Jude, directed by St Jude, has helped in the development (implementation leader, Lima)
	Evidence		Since we demonstrated from the beginning that this was something happening in various parts of the world and that it was working and that there were studies that supported it, I think we didn’t have that much resistance (implementation leader, San Luis Potosi)
	Complexity	At the beginning I think they thought we weren’t going to make it, that it was going to be difficult, that they were going [to] have work overload, that they will have too many patients, many things (nurse director, San Salvador)	I think part of its success is because it’s very simple, you can do it by just looking at the patient (implementation leader, San Luis Potosi)

Abbreviations: ICU, intensive care unit; PEWS, pediatric early warning systems.

Participants described hospital characteristics that influenced the ease of PEWS implementation, including the type of center (subspecialty vs general, academic vs not, and public vs private), size, unit organization, and patient population served. These characteristics determined whether centers had adequate available resources to support PEWS implementation: “We have more scarcity because we are not even part of public health, we are decentralized hospital and sometimes we have to find resources” (nurse director; San Louis Potosi, Mexico). Having inadequate human (staff, specialists) and material (supplies, space, money) resources was a major barrier to PEWS implementation. Staff turnover, the rotation of staff between units or recruitment of new staff, presented another barrier by requiring retraining in PEWS use.

Hospital leaders, such as clinical and administrative directors, served an important role in slowing or promoting PEWS implementation: “There are chiefs that have a lot of enthusiasm and motivation to establish these types of preventive measures and maybe some people don’t have that” (physician director; Lima, Peru). Hospital leaders unconvinced of the importance of PEWS placed barriers to implementation: “The chief of the department is still indecisive...he doesn’t get involved as we would like, it’s difficult, in that sense we have that big barrier” (nurse director; San Louis Potosi, Mexico). However, when supportive, hospital leaders facilitated implementation.

One implementation enabler was a strong culture of safety: “the [staff’s] desire to do something better for the patient, that helped a lot…everything is focused on the benefit of the patient, so the child can go home with good results and no complications” (implementation leader; San Salvador, El Salvador). Similarly, institutional experience with patient deterioration highlighted why PEWS was needed and facilitated implementation: “There was a moral situation…a little girl had just died of a common situation…was not assisted in the general room, died and she could have been saved” (implementation leader; Xalapa, Mexico). Although most participants described a dedication to safety, many also mentioned institutional resistance to change or a medical culture that discouraged interdisciplinary collaboration as a barrier to ongoing PEWS use.

### Characteristics of Individuals Using PEWS

Specific characteristics of clinical staff using PEWS influenced the ease of implementation. These characteristics included resistance to change (stage of change), skill calculating PEWS and measuring vital signs, and other characteristics (Table 2).

Participants at all centers mentioned staff resistance to change as a major barrier to implementation of quality improvement initiatives such as PEWS. A particular challenge was the perception that PEWS would increase workload: “Initially it was very hard…they said they studied already, they were already formed and that it was more work for them” (implementation leader; Xalapa, Mexico). When staff were convinced of the importance of PEWS, however, their conviction became a strong enabler: “I think the attitude of the staff, the commitment, which was the most important thing that made the project possible…they realize this is beneficial and that work becomes easier and faster, they end up accepting it” (foundation administrator; San Salvador, El Salvador).

Other characteristics of clinical staff perceived to impact PEWS implementation included their age, subspecialty training, motivation, and dedication to their work. The staff’s inability to measure vital signs, perform a patient assessment, and correctly calculate PEWS were identified as specific barriers to implementation. With practice, staff who lacked these skills were able to acquire them: “[nurses] forgot to check the pupils…at the beginning we had more than 15% [errors] but then we’re always low, that means the measurements were done correctly” (implementation leader; Xalapa, Mexico).

### Outer Setting

The outer setting was mentioned less frequently than other domains. However, the outer setting, or factors external to the hospital, including health systems and the hospital’s experience with collaboration with other institutions, affected implementation (Table 2).

Participants discussed the effects of health systems on their ability to provide high-quality care and implement interventions such as PEWS, including challenges of being in a resource-limited country with low levels of funding to support improvement initiatives, rigid laws and regulations that affected clinical work, and a small national workforce: “Our country is a poor country, our hospital is a public hospital, we don’t have many resources and it’s difficult to request them” (implementation leader; Lima, Peru). Some participants emphasized that aligning the objective of PEWS with criteria for hospital certification and national priorities served as a major enabler: “We moved forward because this is a new way of work and that was certified not only by the institution but also as quality system of management in the Ministry of Health” (nurse director; Lima, Peru).

In addition to health systems, a center’s experience with collaboration with other institutions affected PEWS implementation. Collaborations included those with other hospitals, professional organizations, and philanthropic foundations on projects related to quality improvement, research, education, and patient care. Participants described collaborative experiences as sources of new improvement ideas for the hospital, which was helpful for PEWS implementation: “The experience you gain when participating and collaborating with projects, that helps” (quality improvement coordinator; San Salvador, El Salvador).

### PEWS Characteristics

Participants reported specific characteristics of PEWS as barriers or enablers to implementation. The characteristics included its origin, strength of supporting evidence, and perceived complexity (Table 2).

PEWS was commonly introduced to a center (PEWS origin) by a clinician who heard about the project from another hospital. The perception that PEWS belongs to one person was a barrier that had to be overcome for successful implementation: “That was very important…from knowing it was my project, my study, to know it was a project for this hospital” (implementation leader; Cuenca, Ecuador). Clarity that PEWS came from an international collaborative also helped win support: “When we heard [the study was being led by] St Jude, this is a world reference in the treatment of cancer in children…we knew we could participate in that project, because it’s a project that comes from a serious staff” (physician director; Cuenca, Ecuador).

All participants were motivated by published evidence supporting PEWS, including its validity, effect on patient outcomes, teamwork, and hospital costs. Many participants were reassured by anecdotal stories of successful implementation from centers with similar resources: “the results were good in other hospitals so that made us think that if other hospitals in the same level could get better, we could definitely get better as well” (implementation leader; Lima, Peru). Seeing how PEWS worked at other centers was particularly valuable to inform local implementation strategies: “it’s very important to know the experiences of other centers because they already made their mistakes and we can prevent those same errors” (implementation leader; Xalapa, Mexico).

Another important PEWS characteristic was its perceived complexity. Initially, staff were concerned that using PEWS would be challenging. Experience, however, alleviated these concerns: “at the beginning it was difficult because we felt like we’re wasting a lot of time…right now is very easy” (nurse director; San Louis Potosi, Mexico). Ultimately, participants believed PEWS was a simple intervention that did not require many resources, facilitating implementation: “a project which doesn’t require a great amount of money and the benefit[s] are huge…we’re not going to invest money, we’re just going to use the resources we have…this influenced a lot in the authorities to support this project” (implementation leader; Xalapa, Mexico).

### PEWS Implementation Process

All hospitals faced barriers. Most barriers, however, were resolved during the standardized implementation process, including adaptation, engagement, piloting, evaluation, and obtaining outside help, allowing all centers to successfully implement PEWS.

All centers planned for PEWS implementation by adapting both the PEWS intervention and their center context (Table 3). Minimal adaptations were made to the PEWS scoring tool, focused on medical terminology and the PEWS algorithm to reflect local processes for escalation of care. All participants, however, described changes to their hospital setting to address barriers from their hospital’s inner and outer settings to support PEWS use. Adaptations included changes to the physical space (posting PEWS information and patient tracking boards), documentation (nursing flowsheet, physician notes), hospital processes (frequency of vital signs, care escalation process), policies, and culture (interdisciplinary teamwork and communication). Challenges making these adaptations delayed implementation: “To implement the [modified nursing] sheet, how to do it…I think those aspects delayed the project…made the implementation process slower” (nurse director; San Salvador, El Salvador).

**Table 3.** Types of Adaptations

Domain	Adaptation type	Example
PEWS Adaptation	Scoring tool	Honestly, there haven’t been too many changes to the original scale, just little changes, maybe of vocabulary (implementation leader, San Salvador)
		We changed oxygen, I think in the US...they have other parameters for oxygen saturation, so, we had to modify that, we are higher [altitude] here so we need it to have 90% saturation…we had to change that (implementation leader, Cuenca)
	Algorithm	Some adaptations I remember for example, on the original [algorithm] if there was deterioration you would call directly to ICU, in our case the pediatrician or the oncologist in shift goes first and he evaluates if he calls ICU depending on the action to take (implementation leader, Lima)
		We did modify the flowchart of action because, for example, we don’t have an intensivist in the hospital 24/7…so the resident in charge of the unit was the one doing the evaluations (implementation leader, San Luis Potosi)
	Other	No, we didn’t make changes to the program, we think it’s perfect, with so many places using it, we just needed to adapt (implementation leader, Lima)
		We just adapted to it, but we didn’t change anything of the program (nurse director, Cuenca)
Site adaptation	Physical modifications	We took PEWS to the entire hospital, we posted posters, logos, in the management documents, boards, pins, we would change the PEWS boards constantly (nurse director, Lima)
		We also implemented the whiteboard with the name of the patients, each of them with their PEWS color, so when you go there you can see how our floor is in general and which child requires more attention (director, Cuenca)
	Documentation	Also, the nursing sheet, we had to make the change official, because it’s a legal document that goes apart from the clinical history (implementation leader, Lima)
		We now have the PEWS scale implemented in a digital way in the medical history, it is now part of the digital medical history (implementation leader, Cuenca)
		We had to modify the nursing sheet, because our sheet is way different from what PEWS requires…we had to see what things to remove from the sheet in order to add the scale (nurse director, San Luis Potosi)
	Hospital processes	We take vital signs 1 time during the day and 2 times during the night. So, make the staff understand that they need to take vital signs more frequently for children depending on their category in the scale (implementation leader, San Salvador)
		We had to reorganize the work, reorganize the teams, try to have more beds available, to improve the discharge process for the patients so we don’t have unnecessary occupied beds. We had to work on that aspect (physician director, Lima)
		First, adaptation, because the staff already had a routine related to the evaluation of the patients. The staff would start the day counting their materials, checking their supplies. When you change their routine, their way of work changes in a drastic way, but the staff adapted to that very fast (implementation leader, Xalapa)
		We did some changes…we asked that when this evaluation is made, there should be a note in the clinical record...and also, the ICU physician should write a note saying what he had found and what actions he took and what modifications he made to the treatment (implementation leader, San Luis Potosi)
	Culture	We also adapted the health care staff...we’re going to do the evaluation with the doctor next to the patient, we’re going to call the coordinator, so the doctor would come to us and we had more contact with the doctors. We also adapted to the doctors’ attitudes, the nursing staff as well, improving the relationships with them, even with the intensivist (implementation leader, Lima)

Abbreviations: ICU, intensive care unit; PEWS, pediatric early warning systems.

All participants also emphasized the need for early engagement of a diverse set of hospital stakeholders through training, informational meetings, and one-on-one conversations to address specific clinical skill deficits and general reluctance to adopt PEWS (Table 4). Important stakeholders included the PEWS implementation leaders, all clinical staff, hospital leadership, and others who supported PEWS adoption and use (champions), including the department of quality and safety, staff educators, and families. Late engagement of these stakeholders resulted in delayed implementation: “Maybe one of the mistakes was not to involve the medical staff, the pediatricians, earlier…it was something unknown for them” (implementation leader; Xalapa, Mexico).

**Table 4.** Components of the Implementation Process

Domain	Theme	Example
Engaging	Staff	I think in all the hospitals that want to implement PEWS; they should involve all the staff possible since the beginning (implementation leader, Cuenca)
		[We presented] the program for all staff including appointed doctors, nursing staff and residents…the program was introduced emphasizing in the role each of us had in the evaluation of the patient (implementation leader, San Luis Potosi)
		The first thing was the information spread on all areas explaining the program. They would give us informational talks, brochures, detailed information about the program, how it was born, how it is implemented...and its goal (nurse director, Xalapa)
	Hospital leaders	We cannot talk with the entire staff, but we can talk about it with the chief pediatrics nursing, chief of pediatrics, chief of residents, chief of emergency department, so that way we can show results and then each chief with their own staff make a revision again, and resolve doubts again, highlight the measures they are taking (implementation leader, San Luis Potosi)
		Motivation was achieved through the socialization of the project because the first presentation to the directors was very important, to count with their support, they give the approval so we can go on (nurse director, San Salvador)
	Champions	They present the project and we try to be a mediator with the authorities from the institute so all the projects can be implemented...the institute committee has had the doors open without obstacles so the PEWS project could develop (research director, Cuenca)
		[The hematologist] convinced the residents working with her saying it was important to detect patients, even though she never got involved in the implementation or in all the processes we did; I think she helped a lot because she convinced the residents that this was something good (implementation leader, San Luis Potosi)
		My role was as a facilitator and communicator of the team boosting the implementation in the oncology service and the link with the authorities so they could give them the necessary tools to develop the program (quality improvement coordinator, San Salvador)
		We even had people...that accepted it so well, they applied it and adapted to it so fast, that even they were the ones motivating their own colleagues saying, look, this helps in this way, if we evaluate the patient this way, if we pay more attention to this, or if we dedicate a little bit more time to this program we will have this benefit (nurse director, Xalapa)
Pilot	Available resources	In the pilot we realized that we didn’t have everything we needed, even though before the pilot we try to have all the equipment, but we always had observations from the assistant staff related to the resources (implementation leader, Lima)
		We learned that we had several weaknesses. One of them was that we needed to…obtain the necessary [vital sign] equipment for the patient’s attention. With the pilot we identified those weaknesses that in certain ways try to solve them (nurse director, San Salvador)
	Adaptation	I learned from the pilot that it’s the right time to arrange everything, it’s the right time to know that for the implementation we must have the nursing sheets ready, the modified sheet, see our algorithm that doesn’t have to change, everything, all our material, our tools. It’s the right time to make any changes we need on PEWS (implementation leader, Lima)
		Also, in the pilot we made certain changes to our nursing sheet that made it difficult for the nurse to compile all the data (implementation leader, Cuenca)
		We discovered the errors we could have, we had to unite the criteria according to the scales we made and were modified according to every institution...we modify them and the final result is the one we are using now (implementation leader, San Luis Potosi)
	Engaging and skill using PEWS	Also, when we trained again the staff, you could know who wasn’t doing things the right way to reinforce their knowledge and explain them better, which was the pilot’s help (implementation leader, Lima)
		Well, we learned a lot of things from the pilot, from improving the techniques of how to take the vital signs, the technique was improved, the speed of the doctor’s response to the patient was improved (research director, Cuenca)
		With the pilot plan we realized that there were too many details we were taking for granted, that we had many errors when it came to direct attention with the patient, from a bad diagnosis to a medical intervention we made. So, it helped us to realize the errors we were making (implementation leader, San Salvador)
	Teamwork	We learned that working in teams was the key for this project’s success (implementation leader, San Salvador)
		I think that’s what the pilot taught us, that we have work in groups, in teams, that we all depend on everyone, that you can’t do it alone, because sometimes at the beginning the leaders wanted to do everything, see the data, see the files, but they realized they couldn’t, that it was a group work (implementation leader, Xalapa)
	Stage of change	We also learned that the project was feasible and it was worth it for the benefit of the patient because we are all benefited, but especially the patient (nurse director, San Salvador)
		We learned not to be so confident, because even a patient who comes in for chemotherapy can deteriorate and reach a yellow PEWS or even red. We learned that we can measure that, that’s something measurable, not subjective, not just say I think he’s not good, I think we won’t make it, to have something objective and measurable that gives me numbers (implementation leader, Xalapa)
	Outcomes	Well, in the pilot what we saw was that the initial mortality rate we had, that I think it was almost 48%, almost 50%, it was a very high mortality, was reduced to more or less 10 points based on the use of the evaluation scale (implementation leader, Lima)
		I think the most important thing is that we made it, that PEWS was recognized as a tool that worked, the pilot showed that it worked and this maybe opened the doors to create the conditions to run it as a quality project from the hospital (physician director, San Salvador)
Evaluation	Evaluation	The fact that the doctor is always checking medical records of critical patients, medical records of patients who died and make an analysis of that information, if there was a detail that could have prevented the outcome, well that was another factor that allow the implementation and the improvement of the service (implementation leader, San Luis Potosi)
		We made surveys among nurses to figure out how they felt regarding the use of PEWS, and we’re very satisfied to have the answers: more than 90% of the nurses felt comfortable with the application of the scale. They feel comfortable with the scale and they recommend the use of the scale for the improvement in attention of all patients (implementation leader, Cuenca)
		The goal when implementing was to measure the errors, to see where we’re failing and make improvements, that is linked with the detection of reds, what was happening with them, what was the evolution of the reds, and what we did right or wrong (implementation leader, Xalapa)
Outside help	Outside help	The hospital in Queretaro helped us; they invited and guided us to work with St Jude...gave us the guidelines to start working; they guided us step by step to start implementing along with St Jude...they gave us tutorial and training (implementation leader, Xalapa)
		But I think that’s been the direct relationship with other institutions, trainings in other institutions, to go and see how the project is working in another institution, all this allowed to strength…especially to be conscious it had applicability (physician director, Lima)
		There were visits from the staff of St Jude, also from the excellence centers, so they could see how the tool was being implemented, mainly to share experiences, to see how we could improve in those things we’re failing (implementation leader, San Salvador)

Abbreviation: PEWS, pediatric early warning systems.

As part of the implementation process, centers were mentored to pilot PEWS, which participants viewed as necessary to identify areas for improvement: “A pilot will always show you the limitations you have and how [to] maximize the benefits you’ve seen” (physician director; Lima, Peru). Lessons learned during the pilot identified additional opportunities to reduce barriers to implementation, including the need to further adapt PEWS, nursing flowsheets, or equipment available to support PEWS use, learning strategies to engage staff in PEWS, the importance of teamwork, increasing skill using PEWS through practice, and seeing the effect of PEWS on patient outcomes (Table 4).

Following the pilot, centers implemented PEWS with ongoing evaluation of PEWS use: “We have to register the PEWS errors…and have meetings to check how PEWS is working, we see if the staff still needs training” (implementation leader, San Louis Potosi, Mexico). These evaluations identified common errors, demonstrated staff satisfaction, and highlighted effect on patient outcomes, further enabling implementation by justifying the ongoing need for PEWS at the centers.

Centers were guided throughout PEWS implementation with outside help from St Jude, other Proyecto EVAT centers, and philanthropic foundations. This assistance informed all components of the implementation process, including obtaining the necessary training and addressing potential barriers from inadequate resources: “The clip from the oximeter is broken…this is a priority for the foundation, we must repair or buy a new one. So, with the equipment maintenance the foundation has been great help” (implementation leader, San Salvador, El Salvador).

## Discussion

We evaluated barriers and enablers to PEWS implementation in resource-limited settings, identifying multiple barriers at the level of the hospital, clinical staff, outer setting, and PEWS intervention. Despite geographic and organizational differences in the characteristics of participating hospitals, barriers were similar across centers, including having inadequate resources, staff resistance to change, components of health systems, and the perceived origin and complexity of PEWS. All centers, however, were able to overcome these barriers during the implementation process using outside help, adaptation, staff engagement, piloting, and evaluation to convert barriers to enablers and successfully implement PEWS. These findings can be used to guide clinicians on strategies to implement evidence-based interventions in resource-limited hospitals to improve patient outcomes. Specific recommendations informed by this work include early engagement of all relevant stakeholders before starting implementation and using a time-limited pilot followed by an evaluation and further adaptation (using quality improvement methods) to proactively identify and address challenges to the adoption and ongoing use of evidence-based interventions in these settings.

In addition, this study provides further evidence for the CFIR framework and supports modifications suggested for use in resource-limited settings.^24^ Similar to prior work,^24^ participants emphasized the importance of the implementation process, including early stakeholder engagement, overcoming resistance to change, contextually piloting the intervention, and evaluating outcomes. Our results, however, identified several differences from what has been reported in high-resource settings. The CFIR describes an intervention as having a rigid core and an adaptable periphery.^22,23^ Although participants mentioned the need to make minor adjustments to PEWS to fit their context, most adaptations occurred in the hospital setting to support PEWS use, including leveraging external collaborations and advocating hospital leadership to overcome existing resource limitations. These findings correspond with evolving evidence that hospitals develop clinical capacity to sustain an intervention during the early implementation process.^34,35^ Factors influencing PEWS sustainability should be explored in future work.

Another unique finding was the importance participants placed on the external origin of PEWS (originating outside their center), the support of the PEWS international collaborative (Proyecto EVAT), and the role of other institutions (St Jude, other Proyecto EVAT centers, and local foundations) in facilitating implementation. Resource-limited hospitals may be more willing to adopt interventions used in high-resource settings and that come with external multicenter, international support. The mentorship and shared experience of other geographically and organizationally similar resource-limited centers served as an enabler to implementation. These findings support recent global efforts to improve outcomes for children with cancer through local empowerment, regional collaborations, and international partnerships,^26,36^ and are a model for the global scale of strategies to support implementation of evidence-based interventions such as PEWS.

### Strengths and Limitations

This work has several limitations. This study included only 5 centers; however, inclusion of in-depth interviews with a variety of key stakeholders adds credibility to our findings. This study is further strengthened by a sample size sufficient to reach thematic saturation, regional and organizational diversity of participating centers, and the variable time required for PEWS implementation. Quantitative analysis of a larger sample comparing center characteristics with time required for PEWS implementation would build on our current findings and should be conducted in future work. Similarly, this study focused on evaluating the implementation of one intervention at pediatric oncology centers, potentially limiting its generalizability to other interventions or other patient populations. The identified barriers and enablers, however, are supported by findings from other contexts,^19,21^ are not specific to PEWS or pediatric oncology, and can broadly inform strategies to implement evidence-based interventions globally. This study was conducted during the COVID-19 pandemic, which affected pediatric oncology care delivery worldwide.^37,38,39^ Although participating centers completed PEWS implementation before March 2020 and experienced barriers independent of the pandemic, sustainability and scale-up of PEWS was likely affected during the pandemic, and this effect should be explored in future work.

## Conclusions

We present the first multicenter, multinational study describing barriers and enablers to PEWS implementation in resource-limited hospitals. The findings suggest that many identified barriers can be converted to enablers through targeted effort during the implementation process. These findings provide guidance on strategies to support implementation of evidence-based interventions to reduce global disparities in patient outcomes.

## Notes

Supplement. **eFigure.** Interview Guide (Back-Translated to English) **eTable 1.** Codebook **eTable 2.** Consolidated Framework for Implementation Research (CFIR) Domains **eTable 3.** Characteristics of Participating Centers
