# Supplementary material for: Assessment of Barriers and Enablers to Implementation of a Pediatric Early Warning System in Resource-Limited Settings
Source: JAMA Netw Open. 2022 Mar 9;5(3):e221547. doi: 10.1001/jamanetworkopen.2022.1547 (PMC8908074; doi:10.1001/jamanetworkopen.2022.1547)
Supplement: Supplement. — eFigure. Interview Guide (Back-Translated to English) eTable 1. Codebook eTable 2. Consolidated Framework for Implementation Research (CFIR) Domains eTable 3. Characteristics of Participating Centers [file jamanetwopen-e221547-s001.pdf]

Supplementary Online Content

Agulnik A, Ferrara G, Puerto-Torres M, et al. Assessment of barriers and enablers to implementation of a pediatric early warning system in resource-limited settings. *JAMA Netw Open*. 2022;5(3):e221547. doi:10.1001/jamanetworkopen.2022.1547

eFigure. Interview Guide (Back-Translated to English)

eTable 1. Codebook

eTable 2. Consolidated Framework for Implementation Research (CFIR) Domains

eTable 3. Characteristics of Participating Centers

This supplementary material has been provided by the authors to give readers additional information about their work.

© 2022 Agulnik A et al. *JAMA Network Open.*

eFigure: Interview Guide (Back-translated to English)

We are conducting a qualitative research study to evaluate the barriers and facilitators in the implementation of the Early Warning Assessment Scale. This study will allow for evaluating the factors that contribute to the successful implementation of PEWS in the centers that participate in the multi-center PEWS project, with the goal of identifying strategies to improve the implementation process in new centers. We are interviewing leaders of PEWS and directors of the hospital.

You are cordially invited to voluntarily participate in this study. Your participation will help us identify ways to improve the implementation of quality improvement programs at a global level. The study consists of conducting individual interviews that will take approximately 30-45 minutes to complete. The interviews are completely confidential, will be recorded as part of the study, and will be transcribed anonymously and deidentified. The purpose of participating in this interview is to improve our understanding of the process of implementation of PEWS and nothing that you say will be associated with your name and it won't affect your employment. Your work relationship with any member of the study team or with St. Jude Children's Research Hospital will not be affected by participating in this study. Although participation in this study is of great help, you have the option to not participate or to stop the interview at any moment. In completing this interview, you agree to participate in this research study.

If you have any questions with respect to this study, please contact the principal investigator, Dr. Asya Agulnik.

To start off, we'd like to know a little more about you with demographic information:

- What's your profession?
- What's your gender?
- How many years have you been working in this center/hospital/institution?
- What role do you currently have in the center/hospital/institution?
- What is your role in Project PEWS? (during PEWS's implementation in your center)

Thanks, now we'd like to start with the interview questions; we're going to talk about quality improvement in your center and questions related to the implementation of PEWS:

1. In general, how would you describe the culture of the personnel in your hospital with regard to patient safety? (how does your institution address patient safety)
    a. To what extent are new ideas adopted or used to bring about improvements in your hospital?
    b. How do you believe that stakeholders, meaning the authorities and influential leaders, respond to initiatives proposed for quality improvement in your hospital?
    c. Before PEWS, did other quality improvement projects exist in your hospital?
        i. Have you ever participated in a quality improvement project before PEWS?
    d. Before PEWS, had your hospital participated in collaborative projects with other centers?
        i. Have you participated in collaborative projects with other centers before PEWS?

2. We're going to talk about the implementation of PEWS in your center. Don't worry if you can't answer some questions. Why did your hospital decide to implement PEWS?

© 2022 Agulnik A et al. *JAMA Network Open.*

    a. Was there a strong need to implement PEWS as an intervention to improve the quality of care in your hospital?
        i. What have you heard about the benefits of PEWS based on evidence from other hospitals?
    b. Was there any external factor (local, state, national) or other considerations (financial, other incentives) that contributed to the decision to implement PEWS in your hospital?
    c. Did you or your team have specific objectives for implementing PEWS in your hospital?

3. Please describe the process of implementation of PEWS in your hospital.
    a. What training did you receive about how to implement PEWS? Was this training sufficient?
    b. How did your hospital plan the implementation of PEWS?
        i. How did you all communicate about PEWS to members of the personnel or departments of the hospital?
    c. What did you all learn from the pilot of PEWS in your center?
    d. What type of changes or adaptations were necessary to facilitate the implementation of PEWS in your hospital? (adaptations to the PEWS program and in your center / work processes for facilitating the implementation)
    e. Beyond the leaders of PEWS, who else had an important role in the implementation?
        i. How did you manage to involve people in your hospital to participate in PEWS?
    f. Was there anyone outside of the hospital who helped with the implementation?
    g. Were there sufficient resources for the implementation of PEWS? If not, did you obtain the necessary resources? How?
    h. What factors facilitated or allowed the implementation of PEWS in your hospital?
    i. What barriers did you or your hospital encounter during the implementation of PEWS?
        i. How did you overcome these barriers?

4. What level of participation have the directors / leaders of your hospital had in PEWS?
    a. What support or actions of the hospital leadership helped to make PEWS a success in your hospital?
    b. Are the leaders of your hospital aware of the accomplishments of the project?
    c. What does the hospital leadership say about PEWS now?

5. What's your opinion about how PEWS currently functions in your hospital, considering the time before COVID?
    a. In the end, was the impact of the implementation of PEWS in your center different from what you expected?

6. What recommendation or advice would you give to a center that wants to implement PEWS?

Do you have any comment about the implementation of PEWS in your hospital that we didn't mention in this interview?

eTable 1: Codebook

Category	Code	Definition
Inner Setting	Hospital Characteristics	Any description of the hospital including organization, type of funding, size, age, etc. Includes description of the pediatric hematology-oncology unit vs other units, general mentions of staffing and workload (if not related to PEWS). Does not include mention of organizations culture (code as "Culture").
	Available Material Resources	The level of resources dedicated to implementation and ongoing use of PEWS, including money, physical space, and materials. This includes monitors and other vital sign equipment as well as PEWS guides or tools, patient boards. Includes mention of how resources were obtained, if mentioning specific resources delegated for this. If the reference is unclear for material vs human resources, code both.
	Available Human Resources	The level of human resources dedicated to implementation and ongoing use of PEWS, including personnel and time, or having enough time dedicated for PEWS training (including providing paid time to attend trainings). If the reference is unclear for material vs human resources, code both.
	Staff Turnover	Any mention of staff turnover, including ration nurses, residents, fellows, physicians, or entry of new staff to the unit. Also includes turn-over of leadership in the hospital. Includes mentions of lack of turnover (staff permanence). If mention of training new staff, double code with "PEWS Training".
	Why PEWS	Why the hospital decided to implement PEWS, including the reason behind the perceived need for this intervention at the center, or how the current situation is intolerable. Frequently involves statements such as "before PEWS (something bad happened to patients)" or "we needed PEWS to improve... in our hospital". Do not double code with "Goals".
	Culture	The approach (norms, values, and basic assumptions) of personnel in the hospital to patient safety and quality, including the desire to do what's best for the patient and provide high-quality patient-centered care, and other elements of the hospital's general culture. Includes mentions employees feeling valued, essential, and psychologically safe to try new methods, including how new ideas for improvements are approached and adopted and how leadership generally respond to new suggestions for improvements. Includes general mentions of how decisions are made and hierarchies.
	Role of Hospital Leaders	Commitment, involvement, and accountability of leaders and managers with implementation and use of PEWS, including their role in PEWS. Includes statements like "the leadership was supportive" and giving mandates to do XYZ with PEWS and general awareness about PEWS.
Characteristics of Individuals	Stage of Change	Willingness, or lack of willingness of individuals or authorities **in the hospital** to gain new skills, accept change, or show interest/enthusiasm for PEWS. Includes mentions of resistance to or embracing using PEWS as part of routine patient care, comments about how useful PEWS is/isn't to patient care and other beliefs about the value of PEWS, perceptions of whether PEWS does/does not increase workload, and recommendations to implement PEWS in other centers. Should only be coded when referring to perceptions or actions of individuals in the organization. May be double coded with 'Sustainability' if mentions both individuals and institutional acceptance of PEWS. Does NOT include knowledge or skill on HOW to use PEWS correctly ("skill with PEWS").

© 2022 Agulnik A et al. *JAMA Network Open.*

	Skill with PEWS	How well individuals in the hospital know how to use PEWS, including mentions of making errors and mistakes and doing it 'well'. Confidence and ability to correctly use PEWS. Includes mention of the current % errors in doing PEWS and ability to take vital signs correctly. Does not include beliefs about PEWS or how willing they are to use it ("Stage of Change")
	Other Characteristics of Individuals	Other mention of characteristics of individuals in the hospital, not related to the other codes. Includes comments about intellectual ability, motivation, age, values, competence, capacity, and interest in learning generally. Includes mentions of human factors. Does not include PEWS Team Leader commitment (code as "PEWS Implementation Leaders")
Outer Setting	Health Systems	Relationship between key system characteristics and implementation, including health systems such as ministry of health, national insurance, government, regulations, country characteristics etc. Example: developing country.
	Experience with Collaboration	Prior experience of the hospital or individuals in the hospital in any projects, research, quality improvement, or other initiatives, involving collaborating with other centers or foundations BEFORE PEWS.
PEWS Characteristics	PEWS Origin	Any mention of where PEWS came from, such as developed by St. Jude, or who 'owns' the project, such as PEWS being the 'project' of one member of the leadership team vs the hospital, and where PEWS started (UNOP, Guatemala, St. Jude).
	Evidence	Any mention of the quality of the evidence, or lack thereof, supporting the implementation of PEWS and its effect on patient care based on experience of **other centers**, published literature, anecdotal stories, and other sources (evidence BEFORE implementation of PEWS at the hospital). Includes mention of cost-effectiveness. The beliefs of others in the organization about how useful PEWS is to them should be coded as "stage of change" and the impact of PEWS on patient outcomes at this center should be coded as "outcomes".
	Complexity	Any mention of the difficulty or ease of implementing PEWS, including intricacy or the number of steps required to implement, the scope, or need to radically disrupt routine process. Includes mentions of feasibility, viability, simplicity.
	Adaptability OF PEWS	Changes made to the PEWS tool, algorithm/flow chart, or system of implementation by the center to adapt it to their unique hospital setting. This includes any general comments about the potential to adapt or change PEWS for use in the center (or lack of ability to do this, i.e., rigidity). Includes direct answers to the question about PEWS adaptation that the speaker perceives as an adaptation of PEWS at their center. This does not include adaptations made by the institution to adapt TO PEWS (code "Site Adaptation")
Process	Site Adaptation	The degree to which PEWS aligns or doesn't align with the existing values and structure of the hospital. This includes mention of changes made in the institution to be able to implement PEWS (such as increased staffing, changing nursing flowsheets, changing the frequency of doing vital signs, shifting schedules to attend training), as well as mentions of the compatibility, or 'fit' between PEWS and the hospital's existing workflows and systems or the values of individuals who work there. This does not include changes made to the PEWS tool, algorithm, or system (code that as "Adaptability OF PEWS").
	Engaging Champions	Other individuals IN the hospital beyond hospital leadership and the PEWS implementation team who had an important role in the implementation of PEWS or served as a barrier or facilitator. This includes Quality Improvement Department staff. 'Individuals who dedicate themselves to supporting, marketing, and driving through the implementation of PEWS and overcome indifference or resistance that it may provoke within the hospital".

© 2022 Agulnik A et al. *JAMA Network Open.*

	Engaging Staff	Mention of how to attract individuals (nurses, physicians) to implementation and use of PEWS through social marketing, role modeling, and other activities. Any mention of communication about PEWS. Exclude statements related to engaging hospital Leadership or Champions (code separately), mention of training (code "Training") or staff participation in PEWS (code "stage of change" or outcomes" depending on context). If trying to decide between engaging staff and leadership (it's unclear) choose this code.
	Training	Any mention of training individuals in the hospital on how to use or implement PEWS. Includes mentions of training 1 and 2 and teaching staff how to perform/use PEWS, or re-teaching staff who make mistakes using PEWS.
	Engaging Hospital Leaders	Mention of engaging, communicating, or training (or lack of communication with) chiefs and directors in the hospital with respect to implementing and using PEWS. Includes unit director, medical director, authorities, nursing chief, and other hospital leadership, "key stakeholders" and how these individuals were engaged or communicated with to participate/support PEWS. Their role or final support (or lack thereof) for implementing/using PEWS should be coded as "Role of Leadership and Authorities." Does not include general statements of hospital leaders being aware.
	Pilot	Any mention of the PEWS pilot or what was learned from it, such as testing PEWS on a small scale and evaluating this experience to make adjustment to the system prior to full implementation.
	Implementation	Mention of carrying out or accomplishing implementation of PEWS, actual 'doing' implementation. Includes mention of how long implementation took (we implemented quickly, or it took a long time) and delays. Do not code pilot, planning ("planning"), quality ("evaluating") or outcomes of implementation. Do not code if referring to the use of PEWS in patient care (state of change).
	Evaluating	The process of obtaining and assessing quantitative and qualitative feedback about progress and quality of implementation of PEWS; data collection, formal (reports, graphs) or informal (discussions, anecdotes, stories of success). Any mention of measurement, analysis of data, errors in use of PEWS, "reds", and discussion, debriefing, or reflection on the implementation process. This is a verb (action). Includes mentions of measuring patient outcomes (tracking deterioration events) but not conclusions/results about patient outcomes (frequency of clinical deterioration events) --"Patient Outcomes." If it includes mentions of evaluating the pilot, code "Pilot." If discussing the outcome of implementation (implementation success), code "Implementation Outcome"
	Outside Help	Individuals or organizations from OUTSIDE the institution who formally influenced or helped implement PEWS. Includes mention of people coming from other centers to train staff, going to other centers for training, experience with HOW other centers implemented PEWS. Includes communication or support given to the center by outside individuals during implementation.

Abbreviations: QI-Quality Improvement; PEWS-Pediatric Early Warning System

© 2022 Agulnik A et al. *JAMA Network Open.*

eTable 2: Consolidated Framework for Implementation Research (CFIR) Domains

CFIR Domain	Description	CFIR Subdomains	Themes Identified
Inner Setting	Attributes of the institution where the intervention is being implemented that influence the success of implementation.	Structural Characteristics, Networks and Communications, Culture, Implementation Climate (Tension for Change, Compatibility, Relative Priority, Organizational Incentives and Rewards, Goals and Feedback, Learning Climate), Readiness for Implementation (Leadership Engagement, Available Resources, Access to Knowledge and Information)	Hospital Characteristics, Experience with QI, Available Material Resources, Available Human Resources, Staff Turnover, Culture, Why PEWS, Role of Hospital Leaders
Characteristics of Individuals	Attributes of the individuals within the organization that influence the success of implementation.	Individual Identification with Organization, Individual Stage of Change, Knowledge and Beliefs about the Intervention, Other Personal Attributes, Self-Efficacy	Stage of Change, Skill using PEWS, Other Staff Characteristics
Outer Setting	Attributes of the setting outside of the institution that influence the success of implementation.	Cosmopolitanism, External Policies and Incentives, Patient Needs and Resources, Peer Pressure	Health Systems#, Experience with Collaboration, Other External Factors
Intervention Characteristics	Attributes of the interventions that influence the success of implementation.	Adaptability, Complexity, Cost, Design Quality and Packaging, Evidence Strength and Quality, Intervention Source, Relative Advantage, Trialability	Origin, Evidence, Complexity, Adaptability of PEWS
Implementation Process	Attributes of the implementation process that influence success of implementation.	Engaging, Executing (Opinion Leaders, Formal Appointed Interval Implementation Leaders, Champions, External Change Agents), Planning, Reflecting and Evaluating	Site Adaptation, Engaging (Staff, Champions, Hospital Leaders, Outside Help), Pilot, Implementation, Evaluating

*adapted from www.cfirguide.org

#as per Means AR, Kemp CG, Gwayi-Chore MC, et al. Evaluating and optimizing the consolidated framework for implementation research (CFIR) for use in low- and middle-income countries: a systematic review. *Implementation science : IS.* 2020;15(1):17.

Abbreviations: CFIR-Consolidated Framework for Implementation Research; QI-Quality Improvement; PEWS-Pediatric Early Warning System; SLP-San Luis Potosi

© 2022 Agulnik A et al. *JAMA Network Open.*

eTable 3: Characteristics of Participating Centers

Center	Hospital Type	Funding Structure	Annual New Pediatric Cancer Diagnoses	PHO beds	ICU type	Average floor nurse:patient ratio (1 nurse for x patients)	Time Required for PEWS Implementation (months)
San Salvador, El Salvador	Pediatric Multidisciplinary	Public	185	24	PICU	6	3
Cuenca, Ecuador	Oncology (adult and pediatric)	Public-Private	75	22	Adult ICU	10	4
Xalapa, Mexico	Oncology (adult and pediatric)	Public	110	27	PICU	4	4
Lima, Peru	Oncology (adult and pediatric)	Public	800	65	Adult ICU	6 to 7	9.9
San Luis Potosi (SLP), Mexico	General (adult and pediatric)	Public	42	0 (integrated with pediatrics)	PICU	6	11.2

Abbreviations: ICU-Intensive Care Unit; PEWS-Pediatric Early Warning System; PHO-Pediatric Hematology Oncology; SLP-San Luis Potosi

© 2022 Agulnik A et al. *JAMA Network Open.*
